# Preoperative systemic inflammatory biomarkers and postoperative day 1 drain amylase value predict grade C pancreatic fistula after pancreaticoduodenectomy

**DOI:** 10.1016/j.amsu.2020.07.018

**Published:** 2020-07-15

**Authors:** Damiano Caputo, Alessandro Coppola, Chiara Cascone, Silvia Angeletti, Massimo Ciccozzi, Vincenzo La Vaccara, Roberto Coppola

**Affiliations:** a Department of Surgery, University Campus Bio-Medico of Rome, Rome, Italy; b Unit of Clinical Laboratory Science, University Campus Bio-Medico of Rome, Rome, Italy; c Unit of Medical Statistic and Molecular Epidemiology, University Campus Bio-Medico of Rome, Rome, Italy

**Keywords:** Pancreatoduodenectomy, Pancreatic cancer, Pancreatic fistula, Postoperative day 1 drains amylase values, Preoperative inflammatory biomarkers

## Abstract

**Background:**

Postoperative day 1-drains amylase (POD1-DA) values are commonly used to predict the risk of pancreatic fistula (PF) after pancreaticoduodenectomy (PD). Perioperative inflammatory biomarkers have been associated to higher risk of complications in different oncological surgeries. Aim of this study was to investigate the utility of the combination of preoperative inflammatory biomarkers (PIBs) with POD1-DA levels in predicting grade C PF.

**Materials and methods:**

From a prospective collected database of 317 consecutive pancreaticoduodenectomies, data regarding POD1-DA levels and PIBs as neutrophil-to-lymphocyte ratio (NRL), derived neutrophil-to-lymphocyte ratio (dNRL), platelet-to-lymphocyte ratio (PLR) were analyzed in 227 cases. P-values <0.05 were considered statistically significant. Receiver operating characteristic (ROC) curves defined the optimal thresholds for biomarkers and drains amylase values and their accuracy to predict PF. Furthermore, the Positive Predictive Value (PPV) was computed to evaluate the probability to develop PF combining PIBs and drains amylase values. Combination of drains amylase and different PIBs cut-offs were used to evaluate the risk of grade C PF.

**Results:**

A POD1-DA level of 351 U/L significantly predicted PF (sensitivity 82.7%, specificity 76%, AUC 0.836; p < 0.001) with a PPV of 76.5% and a NPV of 82.6%.

POD1-DA levels ≥807 U/L significantly predicted grade C PF (sensitivity 72.7%, specificity 64.4%, AUC 0.676; p = 0.004) with a PPV of 17.8% and a NPV of 95.6%.

Notably, this last PPV increased from 17.8% to 89% when PIBs, at different cut-offs, were combined with POD1-DA at the value ≥ 807 U/L.

**Conclusion:**

PIBs significantly improve POD1-DA ability in predicting grade C PF after PD.

## Highlights

• Grade C pancreatic fistula is a major complication after pancreaticoduodenectomy.
• Postoperative day 1-drain amylase levels is a reliable predictor of pancreatic fistula.
• Systemic inflammatory biomarkers and postoperative day 1-drain amylase levels may predict grade C pancreatic fistula.

## 1 Introduction

Pancreaticoduodenectomy (PD) represents the main surgical approach for periampullary tumors. Despite technical improvements in recent years, PD postoperative course is often burdened by not negligible complications rate. Although mortality decreased to less than 5% in high volume centres, morbidity rates remain high [[1], [2], [3], [4], [5]].

Particularly, pancreatic fistula (PF) represents the most common and serious complication after PD and is reported in up to 40% of cases [6].

The International Study Group on Pancreatic Fistula (ISGPF) defined PF as the “output via an operatively placed drain (or a subsequently placed percutaneous drain) of any measurable volume of drain fluid on or after postoperative day 3, with an amylase content greater than 3 times the upper normal serum value”.

Moreover, based on its impact on postoperative course, according to the ISGPF definitions, a grading system allows to identify grade A, B, and C PF [7].

Having no clinical impact on the patient, grade A PF is commonly defined as a “biochemical leake”; among “clinically relevant” PFs (grade B and C), grade C PF is often responsible for critical events that could result in unfavourable outcomes.

Therefore, PF early diagnosis and its management represent a concern for pancreatic surgeons [[8], [9], [10]].

Nowadays, drains amylase levels, at different cut-offs, are used as the only criterion to assess postoperatively the risk for developing PF and may influence patients’ postoperative management [[11], [12], [13]].

However, there is still a lack of preoperative biological markers that can be used to predict the risk of PF and above all grade C PFs.

Recently, several systemic inflammatory biomarkers as perioperative C-reactive protein (CRP), procalcitonin (PCT), neutrophil–lymphocyte ratio (NLR) and derived neutrophil-to-lymphocyte ratio (d-NLR) have been associated with the prognosis of resected patients [14], but, their role in predicting postoperative complications after pancreatic surgery is still debated [[15], [16], [17], [18], [19], [20], [21], [22]].

The aim of this retrospective single-center study was to investigate the utility of the combination of preoperative inflammation biomarkers (PIBs) with postoperative day 1 drains amylase (POD1-DA) levels in predicting grade C PF.

## 2 Materials and Methods

Data from a prospective collected database of 317 consecutive PDs performed at the University Campus Bio-Medico di Roma betwwen 2005 and 2019 have been retrospectively analyzed. Local Ethical Committee approved the study (protocol number 27/19 OSS) registered in ClinicalTrials.com (NCT04361682) and reported according to the STROCSS criteria [23].

Patients undergoing PD for periampullary neoplasms, complete PIBs data and POD1-DA were included in the study. All patients underwent to PD with pancreaticojejunostomy recostruction; two surgical drains were placed at the end of the surgery according to the procedure previously described [24].

No prophylactic octreotide was used in this series, at the moment neither of the pancreaticojejunal anastomosis nor during the postoperative period.

Data regarding PIBs, as full blood count including white blood cell (WBC) count, lymphocytes, neutrophils and platelets counts, neutrophil-to-lymphocyte ratio (NLR), derived neutrophil-to-lymphocyte ratio (d-NLR), platelet-to-lymphocyte ratio (PLR) and POD1-DA levels were collected.

The preoperative NLR and PLR were defined as the ratio of neutrophil count to lymphocyte count (NLR = neutrophils/lymphocytes) and the ratio of platelet count to lymphocyte count (PLR = platelets/lymphocytes), respectively.

The preoperative d-NLR was defined as the ratio of neutrophil count to white blood cell count less neutrophils (d-NLR = neutrophils/leucocytes – neutrophils).

Drains amylase values were measured daily from POD1 by enzymatic assay on the Dimension Vista® 500 System (Siemens healthcare Diagnostics, Germany). PF and its grade have been defined according to the ISGPF guidelines [7]. Eclusion criteria were previous neo-adjuvant treatments and tincomplete laboratory data.

### 2.1 Statistical analysis

Data were analyzed using Med-Calc 18.11.3 statistical package (MedCalc software, Mariakerke, Belgium). Shapiro-Wilk test for Normal distribution was performed to evaluate if data follow the normal distribution.

Receiver operating characteristic (ROC) curves and areas under the curve (AUCs) defined the optimal thresholds for NLR, d-NLR, PLR and drains amylase values and their accuracy to predict PF.

Positive Predictive Values (PPV) and Negative Predictive Values (NPV) were computed to investigate the probability to develop PF combining PIBs and drains amylase values.

Combination of drains amylase and different PIBs cut-offs were used to evaluate the risk of grade C PF. p-values <0.05 were considered statistically significant.

## 3 Results

According to the inclusion criteria, 227 patients were eligible for the study, inclusion criteria are listed in Table 1. Baseline demographic characteristics of the study population are summarized in Table 2. All the variables (age, BMI, drain amylase, NLR, d-NLR and PLR) rejected Normal distribution and median values and interquartile ranges (25th percentile and 75th percentile) (IQR) were considered.

**Table 1:** Inclusion criteria.

Surgery	Pancreatoduodenectomy
Histology	Pancreatic ductal adenocarcinoma
	Distal cholangiocarcinomas
	Adenocarcinoma of the ampulla of Vater
	Duodenal adenocarcinoma
Pre-operative laboratory data	WBC, NLR, d-NLR, PLR
Post-operative laboratory data	POD1-DA

**Table 2:** Demographic characteristics of the study population.

	Study population (227 patients)	
Median age, years	69	
(IQR)	(60.25–75)	
Gender		
Male	131	(57.7%)
Female	96	(42.3%)
Medina BMI, kg/m^2^	24.06	
(IQR)	(22.15–27.59)	

IQR: interquartile range, 25^th^percentile - 75^th^percentile.

Median age of all the series was 69 years (IQR = 60.25–74). Male patients were 131 (57.7%), the median BMI was 24.06 kg/m^2^ (IQR = 22.15–27.59).

Biochemical leak, grade B and grade C PF have been detected in 67/227 (29.5%), 21/227 (9.25%) and 22/227 (9.7%) of the cases, respectively.

**Fig. 1:**
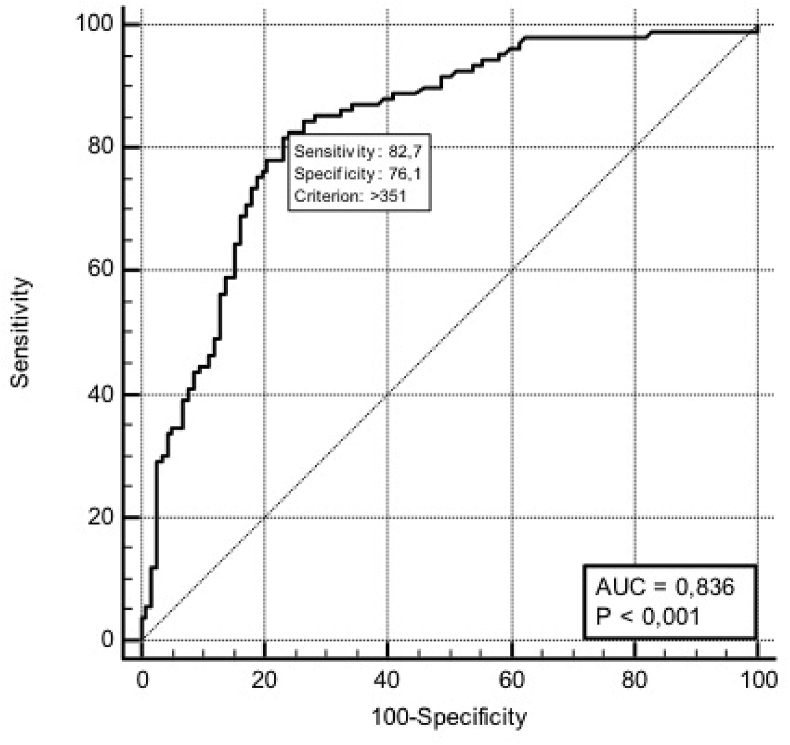
Receiver operating characteristic (ROC) curves of POD1-DA in biochemical leak and clinically relevant PFs. AUC = Area Under the Curve.

ROC curve analysis of POD1-DA has been reported in Fig. 1.

According to the results of ROC curve analysis, a POD1-DA level of 351 U/L significantly predicted both biochemical leak and clinically relevant PFs (sensitivity 82.7%, specificity 76%, AUC 0.836; p < 0.001) (Fig. 1) with a PPV of 76.5% and a NPV of 82.6%.

**Fig. 2:**
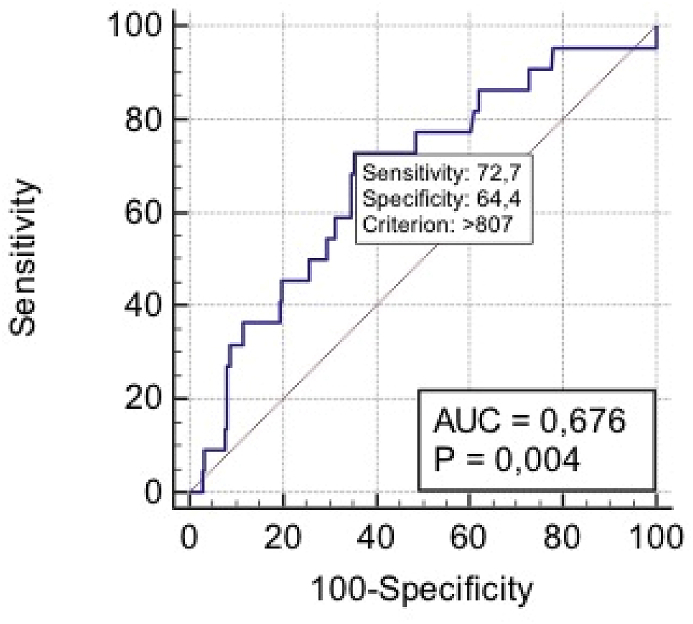
Receiver operating characteristic (ROC) curves of POD1-DA in grade C PF. AUC = Area Under the Curve.

Nevertheless, POD1-DA levels ≥807 U/L significantly predicted grade C PF (sensitivity 72.7%, specificity 64.4%, AUC 0.676; p = 0.004) (Fig. 2) with a PPV of 17.8% and a NPV of 95.6%.

Notably, PIBs at different cut-offs were able to improve PPV of POD1-DA levels to predict grade C PF. More in detail, PPV increased from 17.8% to 89% combining PIBs with POD1-DA at the value ≥ 807 U/L (Table 3).

**Table 3:** Positive predictive values (PPVs) of POD1-DA and PIBs at different cut-offs analysing grade C PF.

	PPV
POD1-DA >807 U/L	17%
POD1-DA >807 U/L + dNLR >3	76%
POD1-DA >807 U/L + dNLR >3 + NLR>3.2	86%
POD1-DA >807 U/L + dNLR >3 + NLR>3.2 + PLR>137	89%

## 4 Discussion

PD represents the standard of care for patients affected by periampullary malignant invasive tumors. Over the last few decades, pancreatic surgery had radically changed. The centralization of pancreatic surgery in high-volume centres reduced PD mortality rate to less than 5%; however, morbidity remains high ranging between 30% and 50% [5].

PF, reported in up to 40% of cases, is the most frequent and challenging postoperative inflammatory complication after PD (23) and is often responsible for other complications and sometimes for mortality even in high volume centres [8].

According to “The 2016 update of the International Study Group (ISGPS) definition and grading of postoperative pancreatic fistula” [25], Grade A PF is defined as “biochemical leak,” since it has any clinical impact.

Grade B and C PFs are defined clinically relevant fistulas; a recent systematic review showed that clinically relevant PF after PD occurred in 12% of patients with a mortality rate of 39% [26].

It is clear that PF early detection and its correct management are crucial for postoperative outcome.

Unfortunately, most of the risk factors for PF are unchangeable; some of them include patient demographic characteristics (e.g. male sex, advanced age, obesity) and phisological or pathological conditions (e.g. pancreatic duct <3 mm, soft pancreatic gland, pathologies other than pancreatic adenocarcinoma or chronic pancreatitis) [27].

On this basis, in the last years different PF risk models have been proposed [[28], [29], [30], [31]]. Unfortunately, none of them seems able to stratify the risk of developing a clinical relevant PF. Moreover, none of them considered the baseline inflammatory status of patients.

Postoperatively, POD1-DA levels are the most used criterion to predict PF risk [31]. However, to date there is not agreement regarding the optimal cut-offs and most of the studies conducted on this topic focused on all grades of PF.

In 2007, Molinari reported how POD1-DA > 5000 U/L was a reliable predictor of PF [11].

Other Authors reported the utility of lower drain amylase levels (e.g. 100 U/L, 350 U/L) [13,33,34].

More recently, Fong demonstrated that the risk of developing PF increases to 31.4% in presence of drains amylase levels in POD-1 higher than 600U/L (p < 0.0001) [35].

However, as stated by Reido-Lombardo, postoperative drains amylase levels alone are not helpful to predict clinically relevant pancreatic fistula [36].

For this reason, other tools that combined with postoperative drains amylase values may predict the risk of developing a clinically relevant and especially a Grade C PF are necessary.

According to other Authors, data reported in the present experience demonstrated that POD1-DA levels is a reliable predictor of the risk of developing biochemical leak and clinically relevant PF after PD. POD1-DA > 351 U/L showed high accuracy in predicting all grade PF.

Neverthelss, POD1-DA >807 U/L was associated to grade C PF with low PPV (17.8%) and high NPV (95.7%). These findings suggest that POD1-DA<807 U/L may identify with high probability patients who will not develop grade C PF. On the other hand, due to the low PPV, POD1-DA> 807 U/L alone is not able to predict grade C PF.

Instead, the combination of POD1-DA> 807 U/L with PIBs at different cut-offs, improving the post-test probability, allowed to identify 89% of these patients. These results suggest that systemic inflammatory status may play an important role in PD postoperative course.

The correlation between cancer and inflammation is still object of investigations; the inflammatory status has been proved to play an important role in promoting carcinogenesis, tumour invasion and metastases [[37], [38], [39]].

In the last decades, PIBs as NLR, d-NLR, PLR, CRP and procalcitonin (PCT) have emerged as predictor of long-term oncological outcomes in different types of tumors [20,[40], [41], [42], [43], [44]].

Neutrophilia and lymphopenia presumably are responsible for an immunosuppressive status that promotes tumor growth and impair the immune reaction against tumor invasion.

Morever, several inflammatory biomarkers, at different cut-offs, demonstrated their utility in predicting postoperative complications in different types of procedures as colorectal [45] and otoryno-laryngeal surgeries. [46,47].

Few and opposing evidences have been reported on the role that inflammatory status may have on pancreatic surgery postoperative outcomes. Some Authors reported that postoperative CRP as a marker for early diagnosis of postoperative inflammatory complications after pancreatic resections. [[48], [49], [50]], while in 2015 Solaini reported a limited utility of inflammatory biomarkers in detection of postoperative morbidity after pancreatic surgery [22].

In Solaini experience, 92 out of 378 patients developed at least one inflammatory complication and 31 (8.2%) a clinically relevant PF. Data regarding preoperative and postoperative white cell blood count (WBC), neutrophils, lymphocytes and serum C-reactive protein (CRP) have been analyzed.

ROC curve analysis showed that the most useful cut-off values for WBC, CRP and NLR were 8.5 10^3^/mL, 188 mg/L and 12.3 respectively. Notably, significant levels were detected preoperatively only for WBC since CRP and NLR demonstrated their ability in predicting inflammatory complications only if dosed in POD 4 and 2 respectively.

Morevoer, multivariate analysis showed how only CPR on POD3, at the optimal cut-off of 272 mg/L, was confirmed as an accurred predictor of PF (diagnostic accuracy 74%, sensitivity 54.5%, specificity 78.5%; PPV 16.88, NPV 94.25).

On the contrary, preoperative NLR ≥2.0 was identified as useful predictor of postoperative morbidity in patients underwent to PD for distal cholangiocarcinoma by Kumamoto [51].

In Kumamoto experience, a total of 84 patients were analyzed; 39 (46.4%) developed grade III or higher postoperative complications according to Clavien-Dindo classification. The most common postoperative complication was anastomotic leakage (36.9%); PF occurred in 27 (32.1%) cases. The Author hypothesized that the altered systemic inflammatory status induces a cytokine storm that leads to microvascular impairment. The unfavourable effect of microvascular impairment on wound healing has been reported by Yao describing the association between high pre-operative NLR levels and vascular thrombosis [52].

In the present experience only preoperative inflammatory markers have been analyzed in order to avoid possible bias due to the alterations of the inflammatory status resulting from the surgical trauma.

According to the best of our knowledge, this is the first study demonstrating the utility that PIBs, cheap and user-friendly tools, have in combination with POD1-DA levels in detecting grade C fistulas.

It is our opinion that the use of simple and inexpensive biomarkers able to predict the risk of severe PF could influence the postoperative management. Patients at higher risk of grade C PF could benefit from early postoperative second levels diagnostic evaluations (eg. Abdominal CT scan) and, if needed, from early invasive procedures (e.g. percutaneous drainage placement) avoiding severe manifestations of PF and organs failure. Furthermore, grade C PF risk stratification could help in the surgical drains postoperative management.

Limitations of our study include its retrospective and single center design and an unselected heterogeneous cohort of patients with all types of histology (pancreatic ductal adenocarcinoma, distal cholangiocarcinomas, adenocarcinoma of the ampulla of Vater, duodenal adenocarcinoma) as well as a long study period.

Further prospective investigations on this topic will be useful to confirm or reject results we have obtained.

## 5 Conclusions

POD1-DA level is an effective predictor of biochemical leak and cinically relevant PF after PD, however its efficacy in predicting severity of this complication was still unproved. Combination of POD1-DA and PIBs, as NLR, d-NLR and PLR, is helpful in the identification of patients who will probably develop grade C PF. If further confirmed, these results may lead to the routine use of the combination of PIBs and POD1-DA as cheap and user-friendly tool for postoperative management of patients submitted to PD.

## Funding

This research did not receive any specific grant from funding agencies in the public, commercial, or not-for-profit sectors.

## Provenance and peer review

Not commissioned, externally peer reviewed.

## Sources of funding

None.

## Ethical approval

This study was approved by the Ethical Committee of University Campus Bio-Medico di Roma (approval number: 27/19 OSS).

## Research registration Unique Identifying number (UIN)

1 Name of the registry: Clinicaltrials.gov
2 Unique Identifying number or registration ID: NCT04361682
3 Hyperlink to your specific registration (must be publicly accessible and will be checked): https://clinicaltrials.gov/ct2/show/NCT04361682?term=NCT04361682&draw=2&rank=1

## Author contribution

**Damiano Caputo:** Substantial contributions to the conception or design of the work, Data interpretation, Drafting the work, Critical revision for important intellectual content.

**Alessandro Coppola:** Substantial contributions to the conception or design of the work.

**Chiara Cascone:** Data Acquisition, Data Analysis, Drafting the work.

**Silvia Angeletti:** Data Analysis, Data interpretation.

**Massimo Ciccozzi:** Data Analysis.

**Vincenzo La Vaccara:** Data Acquisition.

**Roberto Coppola:** Critical revision for important intellectual content.

**All the Authors:** Final approval of the version to be published.

## Guarantor

Chiara Cascone.

Roberto Coppola.

## Declaration of competing interest

None.
